# 4-(3-Chloro­anilino)benzoic acid

**DOI:** 10.1107/S2414314623005989

**Published:** 2023-07-14

**Authors:** Xiaoting Liu, Sihui Long

**Affiliations:** a School of Chemical Engineering and Pharmacy, Wuhan Institute of Technology, Wuhan, Hubei 430205, People’s Republic of China; Goethe-Universität Frankfurt, Germany

**Keywords:** crystal structure, 4-(3-chloro­anilino)benzoic acid, acid–acid dimers

## Abstract

The title compound, C_13_H_10_ClNO_2_, was synthesized by a Buchwald–Hartwig reaction and its crystal structure was investigated for the first time. Crystallization in a variety of solvents led to the discovery of one crystal form. High-quality single crystals were obtained by slow evaporation and the crystal structure was determined by single-crystal X-ray diffraction. The mol­ecules in the crystal structures are highly twisted [the dihedral angle between the aromatic rings is 34.66 (6)°] and pair up to form acid–acid dimers.

## Abstract 2

High quality crystals of 4-(3-chloro­anilino)benzoic acid were grown in aceto­nitrile and its crystal structure was determined by single-crystal X-ray diffraction.

## Graphical abstract

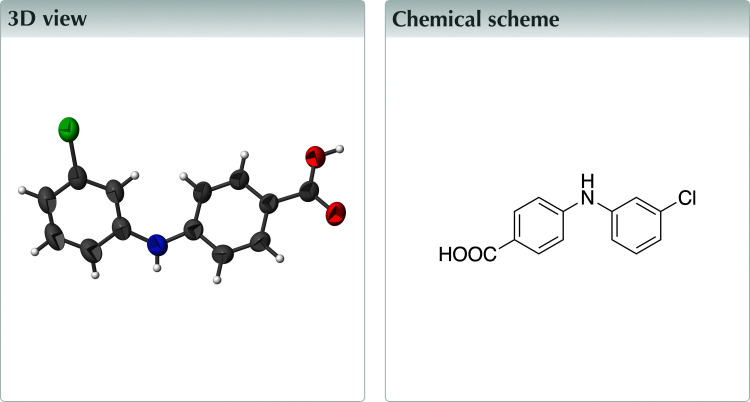

## Structure description

**Figure 1:**
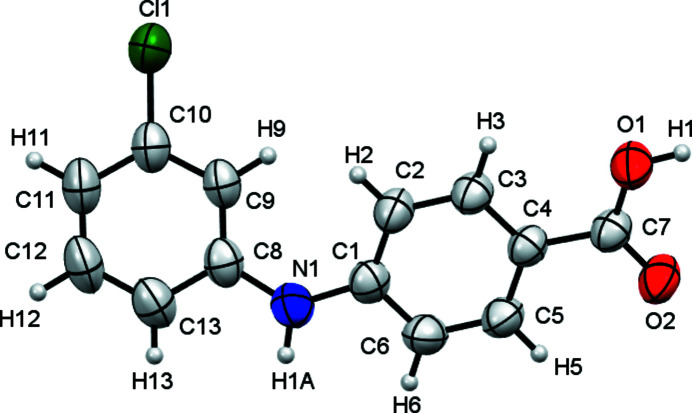
Mol­ecular structure of the title compound generated with *Mercury* (Macrae *et al.*, 2020 ▸), with displacement ellipsoids drawn at the 50% probability level.

**Figure 2:**
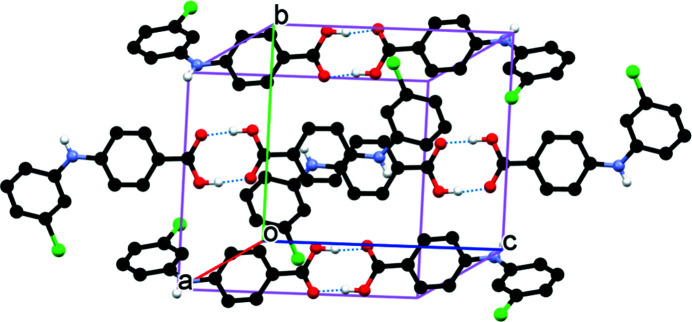
Packing of the mol­ecules of the title compound. For clarity, H atoms bonded to C are omitted.

The title compound (Fig. 1 ▸) was first synthesized by Adeniji *et al.* (2011 ▸). It is a potent and selective aldo-keto reductase AKR1C2 and AKR1C3 inhibitor, which is efficacious in a prostate cancer model and is a potential therapeutic agent for the treatment of castration-resistant prostate cancer (CRPC) (Adeniji *et al.*, 2012 ▸). There is only one mol­ecule in the asymmetric unit of the crystal structure. As a result of the repulsion between the carbon-bonded H atoms *ortho* to NH on both aromatic rings, the mol­ecule is twisted as evidenced by the dihedral angle between the two aromatic rings [34.66 (6)°]. In the crystal, the mol­ecules form acid–acid dimers *via* O—H⋯O hydrogen bonds (Table 1 ▸, Fig. 2 ▸).

## Synthesis and crystallization

**Figure 3:**
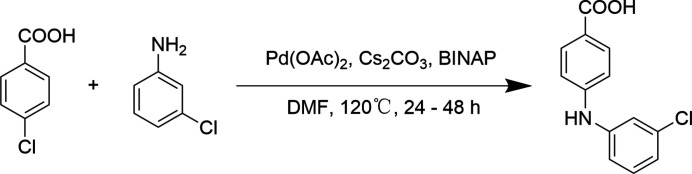
Synthesis of the title compound.

**Figure 4:**
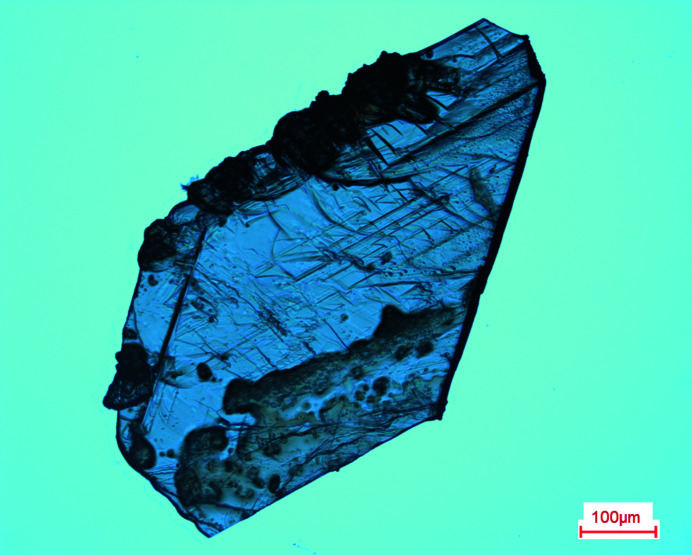
A representative crystal of the title compound.

In this work, the title compound was successfully prepared by a Buchwald–Hartwig reaction (Fig. 3 ▸) (Hou *et al.*, 2015 ▸) using 4-chloro­benzoic acid and 3-chloro­aniline as starting materials. Crystallization was conducted by slow evaporation in avariety of solvents in a clean fume hood. Crystals suitable for single-crystal X-ray diffraction (Fig. 4 ▸) were obtained by slow evaporation of an aceto­nitrile solution.

## Refinement

Crystal data, data collection and structure refinement details are summarized in Table 2 ▸.

## Supplementary Material

Crystal structure: contains datablock(s) global, I. DOI: 10.1107/S2414314623005989/bt4139sup1.cif

CCDC reference: 2280190

Additional supporting information:  crystallographic information; 3D view; checkCIF report

## Figures and Tables

**Table 1:** Hydrogen-bond geometry (Å, °)

*D*—H⋯*A*	*D*—H	H⋯*A*	*D*⋯*A*	*D*—H⋯*A*
O1—H1⋯O2^i^	0.90 (4)	1.78 (4)	2.6359 (19)	160 (4)

Symmetry code: (i) 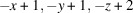 .

**Table 2:** Experimental details

Crystal data	
Chemical formula	C_13_H_10_ClNO_2_
*M*_r_	247.67
Crystal system, space group	Monoclinic, *P*2_1_/*c*
Temperature (K)	288
*a*, *b*, *c* (Å)	10.8865 (2), 10.31441 (18), 11.0885 (2)
β (°)	113.817 (3)
*V* (Å^3^)	1139.08 (5)
*Z*	4
Radiation type	Cu *K*α
μ (mm^−1^)	2.88
Crystal size (mm)	0.15 × 0.13 × 0.09

Data collection	
Diffractometer	Rigaku Oxford Diffraction, Synergy Custom system, HyPix
Absorption correction	Multi-scan (*CrysAlis PRO*; Meyer, 2015 ▸)
*T*_min_, *T*_max_	0.375, 1.000
No. of measured, independent and observed [*I* > 2σ(*I*)] reflections	7087, 2278, 2045
*R*_int_	0.025
(sin θ/λ)_max_ (Å^−1^)	0.633

Refinement	
*R*[*F* ^2^ > 2σ(*F* ^2^)], *wR*(*F* ^2^), *S*	0.047, 0.143, 1.08
No. of reflections	2278
No. of parameters	162
H-atom treatment	H atoms treated by a mixture of independent and constrained refinement
Δρ_max_, Δρ_min_ (e Å^−3^)	0.35, −0.37

Computer programs: *CrysAlis PRO* (Rigaku OD, 2022 ▸), *SHELXS* (Sheldrick, 2008 ▸), *SHELXL2018/3* (Sheldrick, 2015 ▸), *Mercury* (Macrae *et al.*, 2020 ▸) and *OLEX2* (Dolomanov *et al.*, 2009 ▸).

## full crystallographic data

### Crystal data

**Table:**

C_13_H_10_ClNO_2_	*F*(000) = 512
*M**_r_* = 247.67	*D*_x_ = 1.444 Mg m^−^^3^
Monoclinic, *P*2_1_/*c*	Cu *K*α radiation, λ = 1.54184 Å
*a* = 10.8865 (2) Å	Cell parameters from 5783 reflections
*b* = 10.31441 (18) Å	θ = 4.3–77.1°
*c* = 11.0885 (2) Å	µ = 2.88 mm^−^^1^
β = 113.817 (3)°	*T* = 288 K
*V* = 1139.08 (5) Å^3^	Block, clear light colourless
*Z* = 4	0.15 × 0.13 × 0.09 mm

### Data collection

**Table:**

Rigaku Oxford Diffraction, Synergy Custom system, HyPix diffractometer	2278 independent reflections
Radiation source: Rotating-anode X-ray tube, Rigaku (Cu) X-ray Source	2045 reflections with *I* > 2σ(*I*)
Mirror monochromator	*R*_int_ = 0.025
Detector resolution: 10.0000 pixels mm^-1^	θ_max_ = 77.4°, θ_min_ = 4.4°
ω scans	*h* = −13→13
Absorption correction: multi-scan (CrysAlisPro; Rigaku OD, 2022)	*k* = −12→12
*T*_min_ = 0.375, *T*_max_ = 1.000	*l* = −13→14
7087 measured reflections	

### Refinement

**Table:**

Refinement on *F*^2^	Primary atom site location: structure-invariant direct methods
Least-squares matrix: full	Hydrogen site location: mixed
*R*[*F*^2^ > 2σ(*F*^2^)] = 0.047	H atoms treated by a mixture of independent and constrained refinement
*wR*(*F*^2^) = 0.143	*w* = 1/[σ^2^(*F*_o_^2^) + (0.085*P*)^2^ + 0.1891*P*] where *P* = (*F*_o_^2^ + 2*F*_c_^2^)/3
*S* = 1.08	(Δ/σ)_max_ < 0.001
2278 reflections	Δρ_max_ = 0.35 e Å^−^^3^
162 parameters	Δρ_min_ = −0.37 e Å^−^^3^
0 restraints	

### Special details

**Table:**

Geometry. All esds (except the esd in the dihedral angle between two l.s. planes) are estimated using the full covariance matrix. The cell esds are taken into account individually in the estimation of esds in distances, angles and torsion angles; correlations between esds in cell parameters are only used when they are defined by crystal symmetry. An approximate (isotropic) treatment of cell esds is used for estimating esds involving l.s. planes.
Refinement. The positions of all H atoms were obtained from the difference Fourier map. H atoms bonded to N1 and O1 were freely refined. Other H atoms were positioned geometrically with C—H = 0.93 Å and constraioned to ride on their parent atoms with *U*_iso_(H) = 1.2*U*_eq_(C).

### Fractional atomic coordinates and isotropic or equivalent isotropic displacement parameters (Å^2^)

**Table:**

	*x*	*y*	*z*	*U*_iso_*/*U*_eq_	
Cl1	1.08885 (6)	0.18033 (6)	0.52451 (6)	0.0806 (3)	
O1	0.56772 (18)	0.39086 (13)	0.91797 (17)	0.0746 (4)	
H1	0.553 (4)	0.394 (4)	0.992 (4)	0.150 (15)*	
O2	0.53662 (16)	0.60395 (13)	0.90499 (15)	0.0729 (4)	
N1	0.72634 (17)	0.52920 (19)	0.43865 (17)	0.0663 (4)	
C1	0.69502 (16)	0.51613 (18)	0.54768 (17)	0.0547 (4)	
C2	0.69426 (18)	0.39841 (17)	0.60907 (19)	0.0583 (4)	
H2	0.718984	0.322720	0.579069	0.070*	
C3	0.65700 (18)	0.39349 (16)	0.71420 (19)	0.0566 (4)	
H3	0.658316	0.314588	0.755293	0.068*	
C4	0.61751 (16)	0.50519 (15)	0.75936 (17)	0.0511 (4)	
C5	0.61631 (19)	0.62229 (17)	0.69628 (19)	0.0594 (4)	
H5	0.588815	0.697547	0.724422	0.071*	
C6	0.6552 (2)	0.62786 (19)	0.5931 (2)	0.0634 (5)	
H6	0.655065	0.707079	0.552966	0.076*	
C7	0.57147 (17)	0.50230 (15)	0.86682 (18)	0.0542 (4)	
C8	0.80918 (17)	0.4555 (2)	0.39658 (17)	0.0577 (4)	
C9	0.89546 (17)	0.3606 (2)	0.47289 (16)	0.0573 (4)	
H9	0.897922	0.340034	0.555486	0.069*	
C10	0.97786 (19)	0.2970 (2)	0.42486 (18)	0.0612 (5)	
C11	0.9792 (2)	0.3250 (2)	0.3038 (2)	0.0717 (6)	
H11	1.035995	0.281079	0.273724	0.086*	
C12	0.8938 (2)	0.4199 (3)	0.22921 (19)	0.0781 (6)	
H12	0.893432	0.441247	0.147575	0.094*	
C13	0.8084 (2)	0.4843 (2)	0.27326 (19)	0.0710 (5)	
H13	0.750126	0.547161	0.220491	0.085*	
H1A	0.707 (3)	0.599 (2)	0.404 (2)	0.076 (7)*	

### Atomic displacement parameters (Å^2^)

**Table:**

	*U*^11^	*U*^22^	*U*^33^	*U*^12^	*U*^13^	*U*^23^
Cl1	0.0851 (4)	0.0895 (4)	0.0837 (4)	0.0171 (3)	0.0512 (3)	0.0098 (2)
O1	0.1076 (11)	0.0517 (7)	0.0901 (10)	−0.0001 (7)	0.0662 (9)	−0.0001 (6)
O2	0.0978 (10)	0.0509 (7)	0.0929 (10)	−0.0024 (6)	0.0623 (9)	−0.0109 (6)
N1	0.0644 (9)	0.0749 (11)	0.0663 (10)	0.0075 (8)	0.0333 (8)	0.0120 (8)
C1	0.0453 (8)	0.0623 (10)	0.0569 (9)	−0.0007 (7)	0.0212 (7)	0.0001 (7)
C2	0.0618 (10)	0.0515 (9)	0.0731 (11)	−0.0036 (7)	0.0390 (9)	−0.0076 (7)
C3	0.0606 (9)	0.0455 (8)	0.0723 (10)	−0.0027 (7)	0.0358 (8)	−0.0023 (7)
C4	0.0475 (8)	0.0486 (9)	0.0595 (9)	−0.0019 (6)	0.0240 (7)	−0.0047 (6)
C5	0.0633 (10)	0.0486 (9)	0.0721 (11)	0.0066 (7)	0.0332 (9)	0.0002 (7)
C6	0.0666 (10)	0.0548 (10)	0.0750 (11)	0.0087 (8)	0.0351 (9)	0.0136 (8)
C7	0.0550 (9)	0.0467 (9)	0.0659 (10)	−0.0043 (6)	0.0296 (8)	−0.0070 (7)
C8	0.0507 (8)	0.0720 (11)	0.0533 (9)	−0.0104 (8)	0.0242 (7)	−0.0046 (8)
C9	0.0551 (9)	0.0723 (11)	0.0502 (8)	−0.0082 (8)	0.0273 (7)	−0.0039 (7)
C10	0.0599 (9)	0.0727 (11)	0.0584 (10)	−0.0078 (8)	0.0316 (8)	−0.0064 (8)
C11	0.0725 (12)	0.0946 (15)	0.0601 (10)	−0.0076 (10)	0.0392 (9)	−0.0102 (10)
C12	0.0794 (13)	0.1123 (18)	0.0515 (10)	−0.0099 (12)	0.0357 (9)	−0.0001 (10)
C13	0.0648 (11)	0.0932 (15)	0.0552 (10)	−0.0058 (10)	0.0244 (8)	0.0064 (9)

### Geometric parameters (Å, º)

**Table:**

Cl1—C10	1.746 (2)	C4—C7	1.468 (2)
O1—H1	0.90 (4)	C5—H5	0.9300
O1—C7	1.290 (2)	C5—C6	1.372 (3)
O2—C7	1.246 (2)	C6—H6	0.9300
N1—C1	1.389 (2)	C8—C9	1.383 (3)
N1—C8	1.396 (3)	C8—C13	1.396 (3)
N1—H1A	0.80 (3)	C9—H9	0.9300
C1—C2	1.394 (3)	C9—C10	1.380 (3)
C1—C6	1.395 (3)	C10—C11	1.379 (3)
C2—H2	0.9300	C11—H11	0.9300
C2—C3	1.381 (3)	C11—C12	1.374 (3)
C3—H3	0.9300	C12—H12	0.9300
C3—C4	1.392 (2)	C12—C13	1.383 (3)
C4—C5	1.393 (2)	C13—H13	0.9300
			
C7—O1—H1	115 (3)	O1—C7—C4	117.19 (15)
C1—N1—C8	131.05 (18)	O2—C7—O1	122.19 (16)
C1—N1—H1A	113.1 (18)	O2—C7—C4	120.62 (15)
C8—N1—H1A	114.2 (19)	C9—C8—N1	123.47 (16)
N1—C1—C2	124.18 (17)	C9—C8—C13	118.95 (18)
N1—C1—C6	117.08 (17)	C13—C8—N1	117.50 (19)
C2—C1—C6	118.66 (16)	C8—C9—H9	120.4
C1—C2—H2	119.8	C10—C9—C8	119.14 (16)
C3—C2—C1	120.39 (16)	C10—C9—H9	120.4
C3—C2—H2	119.8	C9—C10—Cl1	118.36 (14)
C2—C3—H3	119.6	C11—C10—Cl1	118.92 (16)
C2—C3—C4	120.79 (16)	C11—C10—C9	122.7 (2)
C4—C3—H3	119.6	C10—C11—H11	121.2
C3—C4—C5	118.60 (16)	C12—C11—C10	117.70 (19)
C3—C4—C7	122.12 (15)	C12—C11—H11	121.2
C5—C4—C7	119.22 (15)	C11—C12—H12	119.4
C4—C5—H5	119.6	C11—C12—C13	121.21 (18)
C6—C5—C4	120.76 (17)	C13—C12—H12	119.4
C6—C5—H5	119.6	C8—C13—H13	119.8
C1—C6—H6	119.6	C12—C13—C8	120.3 (2)
C5—C6—C1	120.78 (17)	C12—C13—H13	119.8
C5—C6—H6	119.6		
			
Cl1—C10—C11—C12	−178.04 (17)	C4—C5—C6—C1	0.9 (3)
N1—C1—C2—C3	−177.58 (17)	C5—C4—C7—O1	176.54 (17)
N1—C1—C6—C5	176.83 (17)	C5—C4—C7—O2	−2.8 (3)
N1—C8—C9—C10	−177.07 (18)	C6—C1—C2—C3	−1.1 (3)
N1—C8—C13—C12	176.3 (2)	C7—C4—C5—C6	−178.23 (17)
C1—N1—C8—C9	−10.4 (3)	C8—N1—C1—C2	−28.5 (3)
C1—N1—C8—C13	172.68 (19)	C8—N1—C1—C6	155.0 (2)
C1—C2—C3—C4	1.1 (3)	C8—C9—C10—Cl1	178.49 (14)
C2—C1—C6—C5	0.1 (3)	C8—C9—C10—C11	0.8 (3)
C2—C3—C4—C5	0.0 (3)	C9—C8—C13—C12	−0.7 (3)
C2—C3—C4—C7	177.14 (16)	C9—C10—C11—C12	−0.4 (3)
C3—C4—C5—C6	−1.0 (3)	C10—C11—C12—C13	−0.7 (3)
C3—C4—C7—O1	−0.6 (3)	C11—C12—C13—C8	1.2 (3)
C3—C4—C7—O2	−179.92 (17)	C13—C8—C9—C10	−0.2 (3)

### Hydrogen-bond geometry (Å, º)

**Table:**

*D*—H···*A*	*D*—H	H···*A*	*D*···*A*	*D*—H···*A*
O1—H1···O2^i^	0.90 (4)	1.78 (4)	2.6359 (19)	160 (4)

Symmetry code: (i) −*x*+1, −*y*+1, −*z*+2.
